# Size and lipid modification determine liposomal Indocyanine green performance for tumor imaging in a model of rectal cancer

**DOI:** 10.1038/s41598-019-45038-w

**Published:** 2019-06-12

**Authors:** Shoshi Bar-David, Liraz Larush, Noam Goder, Asaf Aizic, Ehud Zigmond, Chen Varol, Joseph Klausner, Shlomo Magdassi, Eran Nizri

**Affiliations:** 1 Laboratory of Surgical Oncology, Department of Surgery A, Tel-Aviv Sourasky Medical Center and The Sackler Faculty of Medicine, Tel-Aviv University, Tel-Aviv, Israel; 2 Casali Center for Applied Chemistry, Institute of Chemistry, The Hebrew University of Jerusalem, Jerusalem, Israel; 3 Institute of Pathology, Tel-Aviv Sourasky Medical Center, Tel-Aviv, Israel; 4 Research Center for Digestive Tract and Liver Diseases, Tel-Aviv Sourasky Medical Center, and Sackler Faculty of Medicine, Tel-Aviv University, Tel-Aviv, Israel; 5 Department of Clinical Microbiology & Immunology, The Sackler Faculty of Medicine, Tel-Aviv University, Tel-Aviv, Israel

**Keywords:** Mouse, Cancer imaging

## Abstract

Localization of rectal tumors is a challenge in minimally invasive surgery due to the lack of tactile sensation. We had developed liposomal indocyanine green (Lip-ICG) for localization of rectal tumor. In this study we evaluated the effects of liposome size and lipid PEGylation on imaging. We used an endoscopically-guided orthotopic experimental rectal cancer model in which tumor fluorescence was determined at different time points after intravenous (i.v.) administration of Lip-ICG and PEGylated liposomes (PEG-Lip-ICG). Signal intensity was measured by tumor-to-background ratio (TBR), or normalized TBR (compared to TBR of free ICG). Fluorescence microscopy of tumor tissue was performed to determine fluorescence localization within the tissue and blood vessels. Liposomes of 60 nm showed an increased TBR compared with free ICG at 12 hours after i.v. injection: normalized TBR (nTBR) = 3.11 vs. 1, respectively (p = 0.006). Larger liposomes (100 nm and 140 nm) had comparable signal to free ICG (nTBR = 0.98 ± 0.02 and 0.78 ± 0.08, respectively), even when additional time points were examined (0.5, 3 and 24 hours). PEG-Lip- ICG were more efficient than Lip-ICG (TBR = 4.2 ± 0.18 vs. 2.5 ± 0.12, p < 0.01) presumably because of reduced uptake by the reticulo-endothelial system. ICG was found outside the capillaries in tumor margins. We conclude that size and lipid modification impact imaging intensity.

## Introduction

Both laparoscopic and robotic minimally invasive surgery (MIS) are increasingly utilized for the treatment of rectal cancer^1^. Recent clinical trials highlighted improved short-term post-operative outcomes in terms of blood loss, pain and recovery, while the long-term outcomes were not inferior to the open technique^2–4^. However, the application of MIS may decrease the ability to localize the tumor due to the loss of tactile sensation. This, in turn, can impair oncological borders in the case of a too limited resection, or impair post-operative quality of life in the case of a too extended one. In addition, wide surgical margins can lead to lower anastomosis and, consequently, to the performance of a diverting ileostomy. Due to their reduced size the problem of tumor localization is even greater in early-stage tumors and in those that had been responsive to neoadjuvant treatment. A possible solution is the utilization of endoscopic marking, although localization of the tumor may still not be straightforward^5–7^. Furthermore, endoscopy is an invasive approach, which adds technical complexity and costs to the operation.

Indocyanine green (ICG) is a near infra-red dye, currently used in various intra-operative settings, such as cholangiography^8^, determination of anastomosis perfusion^9,10^, and tumor localization^11^. Its optical properties enable penetration of human tissues up to 10 mm without auto-fluorescence^12,13^. We had earlier described the use of liposomal ICG (Lip-ICG) for imaging of rectal tumors, and shown its superiority relative to free ICG^14^. We assumed that the retention of Lip-ICG in the tumors is based on the enhanced permeability and retention effect (EPR), which describes the selective leakiness of specific-size molecules at fenestrated peritumoral capillaries and their retention in the tumor due to its abnormal lymphatic drainage^14^.

Various parameters, however, may affect imaging by liposomes. Particle size is known to affect circulation time, due to opsonization by the reticulo-endothelial system (RES) and by tumor accumulation^15,16^. Modification of the lipid moiety of the liposome by linking of polyethylene glycol (PEGylation) is known to increase the half-life of liposomes in the blood, and to reduce their uptake by the RES^17,18^. While developing Lip-ICG for the intra-operative imaging of ureters, we had also shown that various liposomal parameters, such as size and loading dose, influenced *in-vivo* imaging^19,20^.

In the present research we wanted to determine the effect of particle size and PEGylation on liposome imaging performance. In addition, we sought to detect the location of ICG within the tumor by fluorescence microscopy.

## Results

### Induction of rectal tumors in mice

**Figure 1:**
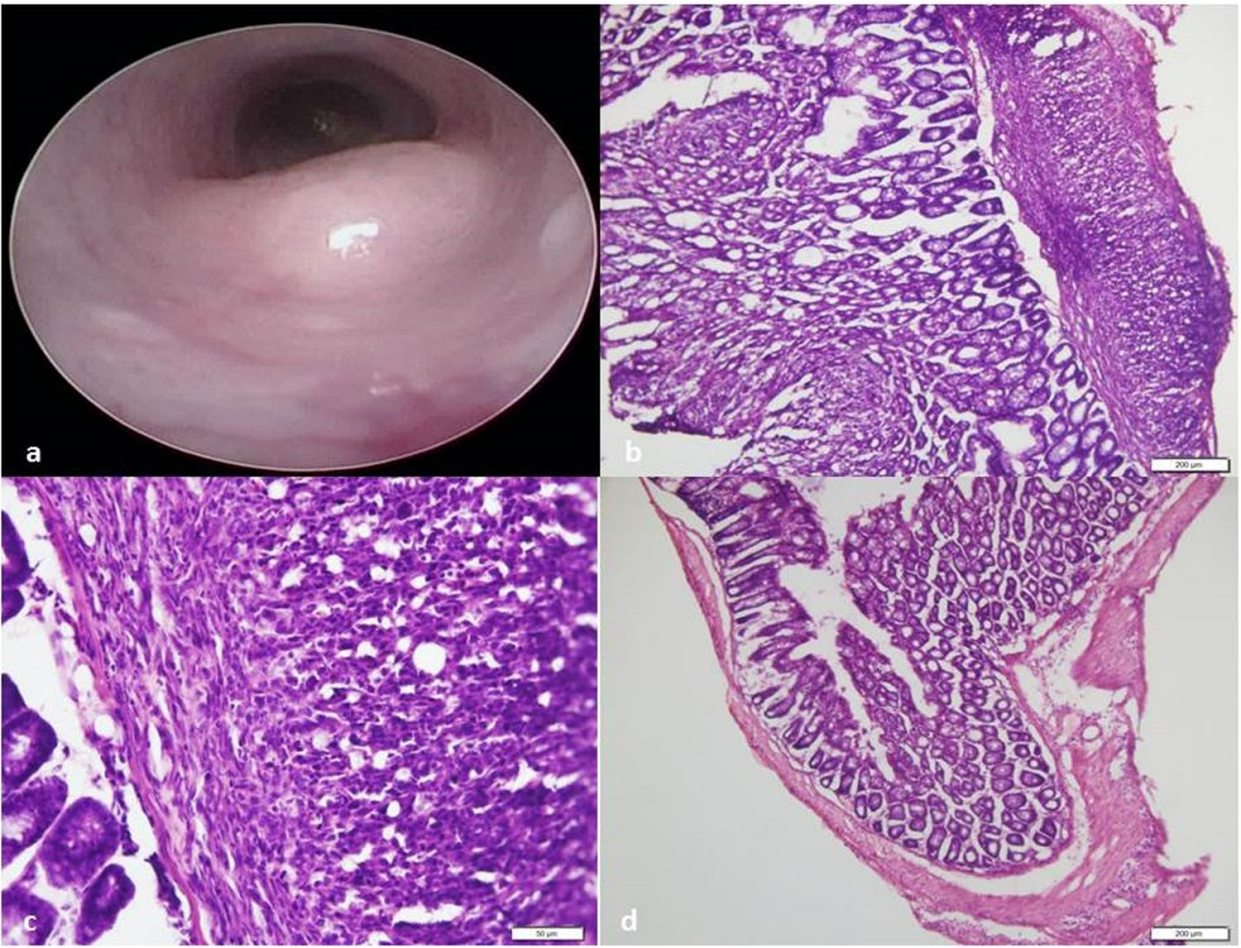
Endoscopic and histologic appearance of the rectal tumor model used in imaging experiments. (**a**) Endoscopy performed 2 days before fluorophore inoculation, showing the tumor protruding at the rectal lumen. (**b**) Histologic section showing an intact mucosa (left) and the tumor cells in the serosa (right) (x100). (**c**) Higher magnification of B (x400). (**d**) Distal normal colon shown for comparison (X100).

The mice were endoscopically injected with 5 × 10^4^ MC38 cancer cells as detailed in the materials and methods section^21^. They were monitored until the tumors obstructed 30–50% of the rectal lumen, as verified by endoscopy (Fig. 1a), after which imaging experiments were conducted. Histological sections of the tumors showed the submucosal location of the tumors induced by this technique (Fig. 1b). Figure 1c shows higher magnification of the tumor cells that display dysplasia and a disorganized structure (non-glandular), consistent with high-grade tumor. In comparison, Fig. 1d shows an adjacent normal bowel wall.

### Effect of liposomal size on tumor imaging

**Figure 2:**
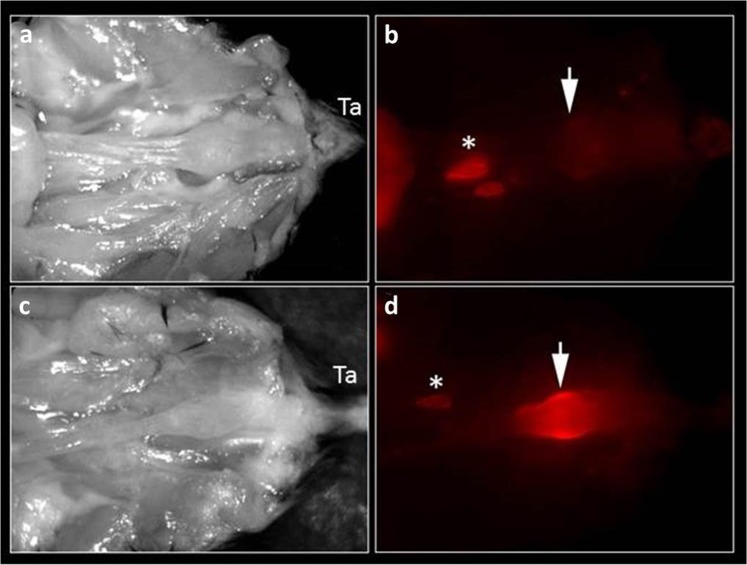
Tumor imaging with liposomal ICG (Lip-ICG) at 60 nm. The mice underwent a median laparotomy during which the urine bladder and genitalia were resected in order to expose the rectum; Ta, tail. (**a**,**b**) Free ICG; (**c**,**d**) Lip-ICG. The arrows indicate the tumor, and the asterisks indicate draining peri-caval lymph nodes. Shown is one representative mouse from each group, out of three experiments, n = 12 for free ICG and n = 20 for Lip-ICG. The signal intensity was determined 12 hours after i.v. injection.

**Figure 3:**
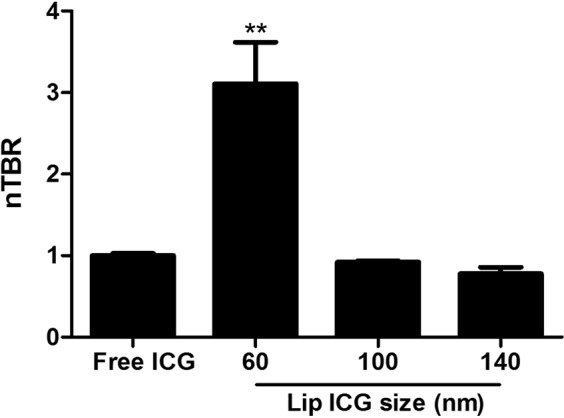
Effect of liposome size on tumor imaging. Different liposome sizes were compared with free ICG, 12 hours after i.v. injection. The results are the summary of two experiments, n = 4 for each size. nTBR = normalized TBR (relative to TBR of free ICG). **p = 0.006.

**Figure 4:**
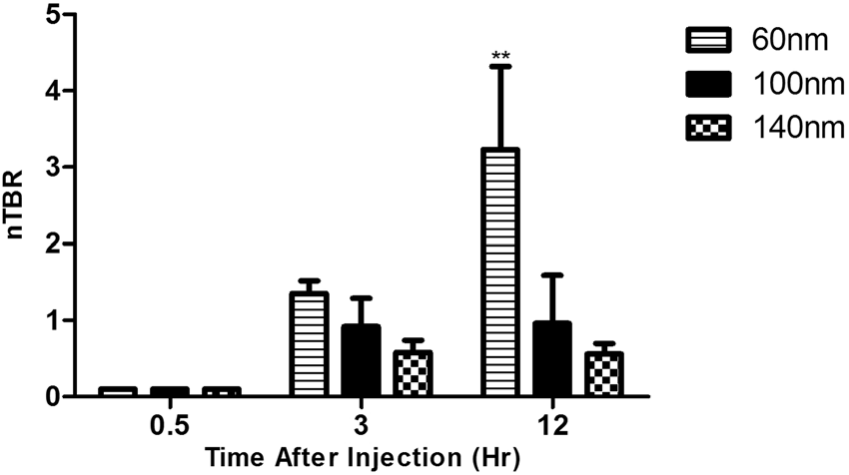
Time course of signal intensity of different size liposomes. The results summarize two experiments, n = 4 for each time point. nTBR = normalized TBR (relative to TBR of free ICG) **p = 0.006.

Liposomes at 60 nm were found to enable tumor imaging that was superior to imaging by free ICG 12 hours after i.v. inoculation (Fig. 2). Since imaging with liposomal carriers is dependent upon extravasation from peritumoral capillaries and accumulation in the tumor, their size may be a relevant factor in determining signal intensity. We tested different liposomal sizes at the same time point, as shown in Fig. 3. While 60 nm liposomes yielded an nTBR (relative to a TBR of free ICG) of 3.11 ± 0.11 (p = 0.006 vs. free ICG), 100 and 140 nm liposomes had nTBRs of 0.98 ± 0.02 and 0.78 ± 0.08, respectively (p = 0.99 and 0.86 vs. free ICG, respectively). Due to the possibility that imaging kinetics may change with liposome size, we tested the various sizes at different time points. Tumor imaging performed 30 minutes after liposome injection did not yield any signal (Fig. 4) for any of the sizes. At 3 hours post-injection no specific imaging was obtainedand the nTBR for 60 and 100 were 1.35 ± 0.17 and 0.92 ± 0.37, while the nTBR was significantly reduced for 140 nm, nTBR = 0.58 ± 0.16 (p = 0.009 vs. 60 nm). At 12 hours the maximal nTBR was again obtained with the 60 nm liposome, while the 140 nm size was significantly reduced (nTBR = 0.56 ± 0.14 vs. 60 nm, p = 0.03). The fluorescence signal at the tumor vanished after 24 hours, but it remained in animal’s gallbladder and partially in the liver. Signal intensity from the liver, spleen and kidney were comparable with all liposome sizes (see Supplementary Fig. S1).

### Effect of liposomal modification on tumor imaging

**Figure 5:**
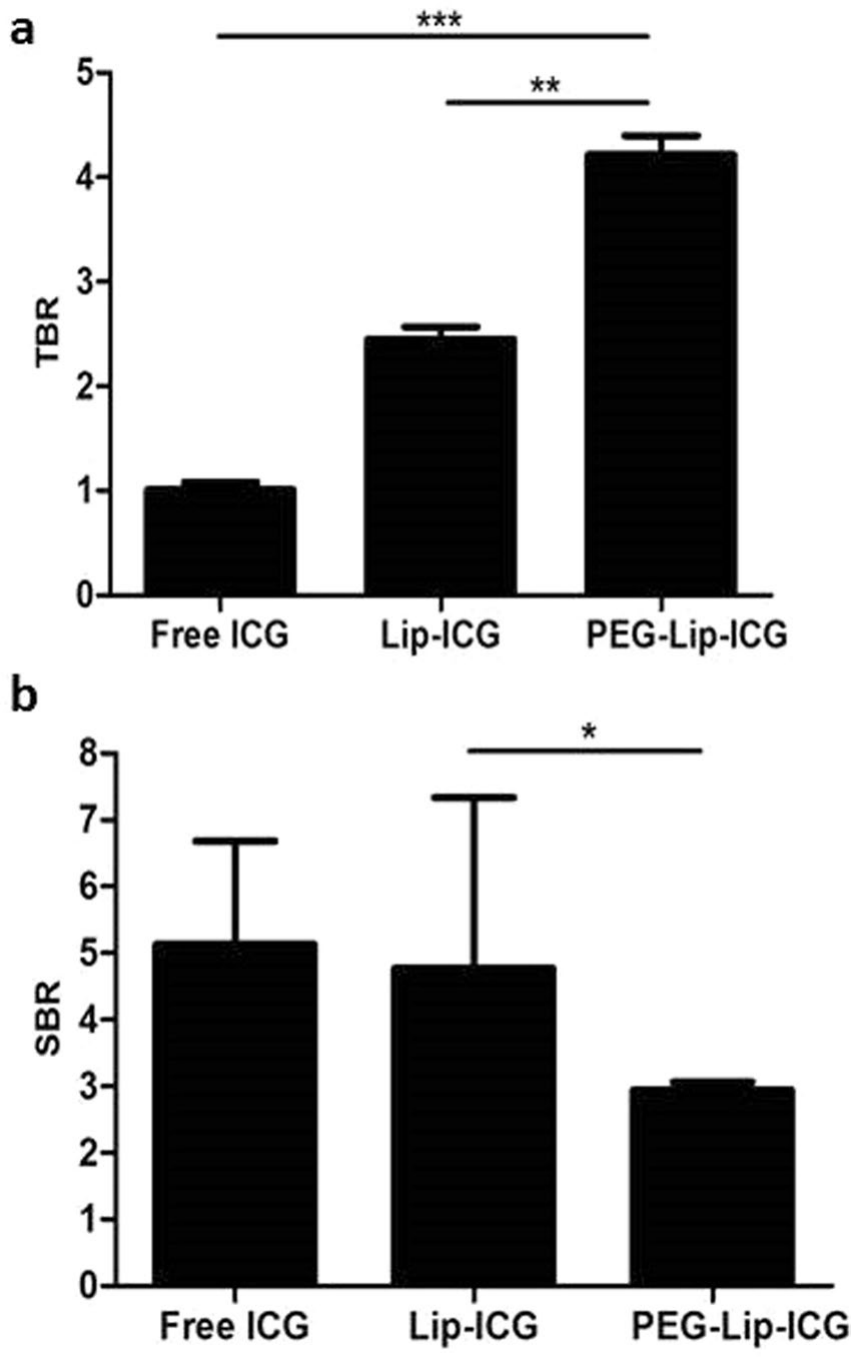
Effect of liposome PEGylation on imaging. (**a**) PEGylated liposome (PEG-Lip-ICG) yielded a significantly higher tumor to background ratio (TBR) when compared with Lip-ICG (4.2 ± 0.18 vs. 2.5 ± 0.12, respectively, p < 0.01). (**b**) Uptake by the reticulo-endothelial system (determined by splenic uptake of the fluorophore, measured as signal-to-background ratio, SBR) showed decreased uptake of PEG-Lip-ICG compared with Lip-ICG (2.95 ± 0.12 *vs* 4.8 ± 2.3, respectively, p < 0.05). Results from one experiment, n = 5 for each group. The signal intensity was determined 12 hours after i.v. injection.

As mentioned above, PEGylation of the phospholipid moiety of the liposome increases its circulating time, mainly due to prevention of its uptake by the RES. We therefore prepared PEG-Lip-ICG and tested their imaging properties. Measurements of the PEG-Lip-ICG revealed their size to be about 80 nm. Figure 5a shows that PEG-Lip-ICG has a TBR of 4.2 ± 0.18 *vs*. 2.5 ± 0.12 for the unmodified liposome (p < 0.01). Analysis of fluorophore uptake by the RES (fluorophore intensity in the spleen) (Fig. 5b) showed that fluorescence was decreased by about 40% (from 4.8 ± 2.3 to 2.95 ± 0.12, p < 0.05) after PEG-Lip-ICG injection.

### ICG localization in the tumor microenvironment

**Figure 6:**
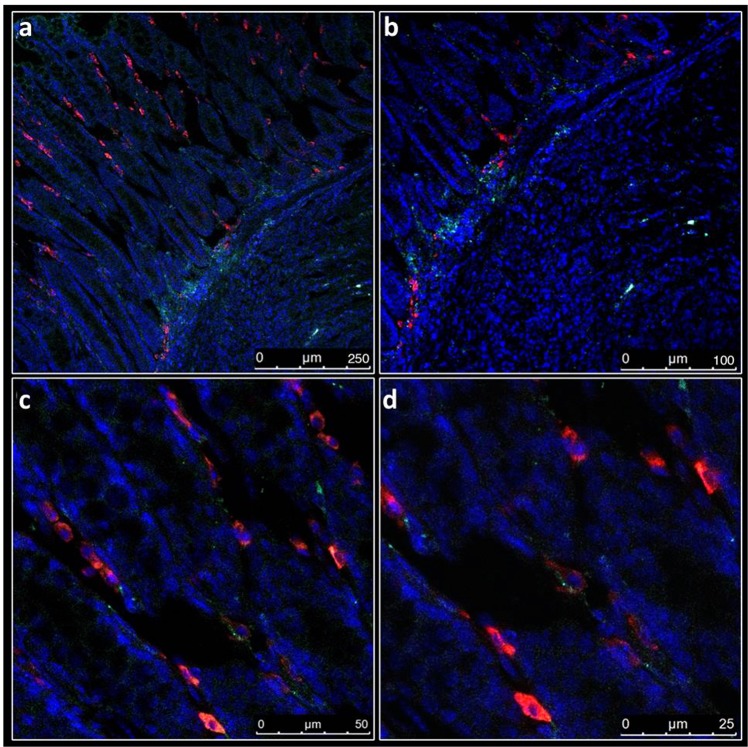
Fluorescence microscopy of ICG in tumor and normal tissue. (**a**) ICG (green) extravasated blood vessels (red, anti-CD31; blue DAPI) at the tumor margins. It may also diffuse into the tumor, as shown in lower left, x200 magnification. (**b**) Higher magnification (x350) of A. (**c**) In normal mucosa ICG is seen in the blood vessels (x600). (**d**) Higher magnification of C, showing ICG retained in capillaries (x1000). Mice were i.v. injected with PEG-Lip-ICG and signal intensity determined after 12 hours.

We analyzed tissue sections of tumor and normal adjacent bowel wall by fluorescence microscopy in order to investigate the localization of the fluorophore within the tumor microenvironment. Mice were injected with PEG-Lip-ICG under the same conditions as described above, and signal intensity was determined after 12 hours. The endothelial cells were marked by anti-CD31. The normal appearing mucosa and tumors were differentiated morphologically by nuclear staining (DAPI) (see also Fig. 1). Figures 6a,b demonstrate ICG as being located outside the peri-tumoral capillaries, although its diffusion into the tumor is also observed in Fig. 6a. ICG is retained in blood vessels in normal mucosa as depicted in Fig. 6c,d.

## Discussion

In this study we described the effects of liposomal size and lipid moiety modification on signal strength in an experimental murine model of rectal cancer. We found liposomes of 60 nm to be optimal for imaging and that PEGylation further enhanced their efficacy.

For future clinical application of Lip-ICG an interval of 12 hours between administration and imaging mandates that imaging should be planned before surgery. Ideally, Lip-ICG should enable immediate, or at least relatively rapid, intra-operative imaging. Hence, we tested larger-size liposomes, which were presumed to be potentially more specific to the tumor due to their inability to extravasate in normal capillaries. However, the signal obtained by the 100 and 140 nm liposomes at any time point was not statistically comparable to that obtained by free ICG. Since the signal intensity of other organs of these liposomes was comparable to that obtained with 60 nm liposomes; the reduced signal emerged as a site (tumor)- specific phenomenon. We assume that increased liposome size prevents the liposomes from extravasating the peri-tumor vessels. Accordingly, as seen in Fig. 4, the signal intensity increased for the 60 nm liposomes during the first hours after injection, but did not change for the larger liposomes, possibly due to ICG accumulation in the tumor for the smaller liposome. In addition, liposomes of 140 nm performed worse than smaller ones even in early time points after administration. However the possibility of altered release kinetics of larger particles could not be excluded. A decrease of transvascular transport with an increase in liposomal diameter has been reported, although in the range of 400–600 nm^22^. It is possible that capillary fenestration sizes vary among different experimental models.

PEGylation of liposomes is a well-described modification of the lipid moiety and is known to increase circulation time. However, it may also increase particle size, as had occurred with our liposomes, which increased from 60 nm to 80 nm. Although the 100 nm liposomes did not have any signal in our system, the increase in liposome size to 80 nm did not offset the benefits associated with PEGylation.

Imaging with liposomes yielded a rim-like fluorescence macroscopically, and this pattern was also evident at the microscopic level, in which ICG was located in peritumoral capillaries, but not within the tumor itself. The lack of intratumoral distribution of liposomes is a well-described limitation of liposome-based therapy. It is attributed to the location of the abnormal, leaky vessels in the tumor periphery and to the increased intratumoral, interstitial pressure which limits passive liposomal diffusion^23^. However, while the peripheral distribution of liposomes may impair their therapeutic efficacy as drug carriers^24^, it does not impair tumor imaging, in which tumor extent and border delineation are required. Theoretically, decreasing liposome size below 60 nm could improve its intra-tumoral diffusion and possibly enhance intratumoral distribution. However, we did not attempt it because the 60 nm liposome, but not a smaller size, enabled ureteral imaging, as we had shown elsewhere^19^. Given that ureteral identification is an important step in rectal surgery, we reserved the possibility of applying Lip-ICG to these two aims. Our results showed that although ICG was located in the tumor periphery after liposomal injection, the tumor could still be identified. It is important to note that we detected the presence of ICG in the tissues, but not of liposomes. The data presented herein do not define whether the fluorophore remained within the liposomal carrier, or whether the liposomes had already been disaggregated inside the tissue.

This study has several limitations. First, since imaging with liposomes depends on the properties of the tumor microenvironment, specifically abnormal tumor capillaries, it may vary in different experimental models. We used an orthotopically induced model, in which the tumor microenvironment was the bowel. However, genetically engineered models should be used to further substantiate our findings. In addition, other liposomal properties, such as liposome electrical charge and length of PEG chains^25–27^, could also be modified to improve tumor imaging. Lastly, the application of Lip-ICG in small animals is different from its use in large animals and humans, in which fascia and fat form thicker layers that may impair imaging quality.

To conclude, we show here that liposomal ICG can be utilized for rectal tumor imaging. Whereas increasing liposomal size had impaired imaging performance, PEGylation of the carrier improved imaging, probably by decreasing its uptake into the RES system. The fluorophore was present in the tumor periphery, outside the peritumoral capillaries. Further research in different experimental models should be carried out to exploit the benefits of this technology in other tumors.

## Methods

### Liposomal ICG preparation

The liposomal ICG was prepared by the following procedure: 18.6 gr of sucrose (9.3%), 10 gr of lecithin, Phospholipon S75 (Lipoid, Steinhausen, Switzerland) (5%), and 171.4 gr of phosphate buffer with concentration of 2 mM (85.7%), were mixed by a magnetic stirrer at 40 °C under nitrogen until homogeneous dispersion was obtained. The liposomal dispersion was sonicated in an ice bath under nitrogen, by an ultrasonic cell crusher SKL-750 (SYCLON), with a probe diameter of 1.1 cm, for 5/20/100 min for obtaining 140/100/60 nm liposomes size respectively, with short breaks of a few seconds in order to add ice to the bath. The sonication conditions were: amplitude: 80%, ON- 2 sec, and OFF- 1 sec. Distilled water (99.52 gr) was added to 480 mg of ICG powder (Sigma-Aldrich, Rehovot, Israel), to obtain an ICG solution in a concentration of 6.2 mM. The powder was mixed with water, and the vial was covered by aluminum foil until the ICG was fully dissolved. Then, 190 gr of liposome dispersion were mixed with 38.02 gr of ICG solution 6.2 mM, covered with aluminum foil, and mixed by a magnetic stirrer over night at room temperature (RT). The liposomal ICG dispersion was cooled by liquid nitrogen to freezing point and then lyophilized for 24–48 hours, (FREE ZONE 2.5, Labconco, MO, USA) at −43 °C, for less than 1 mBar. The powder was dispersed in phosphate buffer prior to the animal experiments. The ICG loading content for liposomal ICG is 1.92% (w/w).

PEGylated liposomes (PEG-Lip-ICG) were prepared by the following procedure: 1.74 gr of sucrose (9.3%), 0.9355 gr of PEG2000 (Lipoid) (5%) and 16.0351 gr of phosphate buffer concentration of 2 mM (85.7%), were mixed by a magnetic stirrer at 40 °C under nitrogen, until a homogeneous PEG2000 dispersion was obtained. Simultaneously, 6.99 gr of sucrose (9.3%), 3.742 gr of lecithin, Phospholipon S75 (5%) and 64.43 gr of phosphate (2 mM) (85.7%) were mixed as described above. Both PEG2000 and liposomes dispersion were mixed until a homogeneous dispersion was obtained. The liposomes-PEG dispersion was sonicated as described above, after which 70 gr of liposomes-PEG dispersion was mixed with 14 gr of ICG solution (6.2 mM) and prepared as described above. The liposomal-PEG-ICG dispersion was lyophilized and then dispersed in phosphate buffer 2 mM before the animal experiments. The ICG loading content for PEG-Lip-ICG is 2.14% (w/w).Prior to the *in-vivo* experiments, liposomal size was measured after lyophilization by Zetasizer Nano-S (Malvern Instruments, Worcestershire, UK) as described elewhere^28^. For 140 nm liposomes: Z Average: 135.3 nm, peak 1: 154.2 nm, 82.6%, peak 2: 26.45 nm, 16.9%, peak 3: 4928 nm, 0.5%, polydispersity index (PDI): 0.270. For 100 nm liposomes: Z Average: 104.4 nm, peak 1: 98.55 nm, 95.4%, peak 2: 21.82 nm, 4.4%, peak 3: 4848 nm, 0.2%, PDI: 0.277. The measurements of these liposomes did not change after the addition of ICG. For 60 nm liposomes: Z Average: 73.06 nm, peak 1: 58.91 nm, 100%, PDI: 0.219. For 60 nm Lip-ICG after lyophilization: Z Average: 90.41 nm, peak 1: 63.48 nm, PDI: 0.272. For 60 nm PEG-Lip-ICG- after lyophilization and 5 min of sonication: Z Average: 95.79 nm, peak 1: 83.34 nm, 100%, PDI: 0.222. ICG concentration of Lip-ICG and PEG-Lip-ICG is 1.92% (w/w) and 2.14% (w/w), respectively.

In order to ensure that there was no free ICG in the lyophilized powder, the liposomes-ICG dispersion, (after lyophilization), was filtered by 300,000, 100,000, 50,000 and 30,000 D filtration tubes (VIVA SPIN, 10 min at 80 rpm, Centrifuge CN-2200, MRC), and the aqueous phase of the dispersion was collected.The ICG concentration in the filtrate was determined by fluorescence measurement at 780 nm (Cary Eclipse Fluorimeter, 1 cm quartz cell and a scan rate of 600 nm/min). The excitation slit was fixed at 5 nm and the emission slit was fixed at 5/10/20 nm. The fluorescence values obtained in the measurements were 0 for all the filtrates in all slits, which indicates that the filtrates did not contain free ICG.

### Animals

The animal study protocol was approved by the Tel-Aviv Sourasky Medical Center Animal Care and Use Committee (no. 7n-4-15), and the procedures followed were in accordance with the institutional guidelines. The 7–8 weeks old C57Bl mice were purchased from Harlan laboratories (Rehovot, Israel) and kept in the animal facility of the Tel-Aviv Sourasky Medical Center. The mice had free access to food (a standard diet) and water, and were maintained on a 12/12-h automatically-timed light/dark cycle. The mice were endoscopically injected with 5 × 10^4^ murine MC38, colon cancer line cells, as previously described^21^. The animals were observed daily for clinical signs, and colonoscopy to stage tumor was done at 14 days post inoculation. Imaging experiments were conducted when the rectal tumors occupied about 30–50% of the rectal lumen, as determined by an experienced endoscopist.

### Imaging experiments

The mice were i.v. inoculated (tail vein) with 8 mg/kg^20^ of free or liposomal ICG of different sizes and imaging assessed at various times points as indicated. For calculation purposes the weight of each mouse was considered 25 gr. And hence each mouse received 0.2 mg of ICG. For specific calculation of each formulation, see Supplementary Table S1. Before imaging experiments, mice were anesthetized and subjected to laparotomy. The bowel was taken out of the imaging field and covered with black paper in order to avoid bowel stool fluorescence. Mice genitalia and urine bladder were resected to expose the rectum. Whole body fluorescence was measured by CRi maestro (Cambridge Research & Instrumentation, Hopkinton, MA, USA), and organs fluorescence was measured by FusionFx7 biomolecular imager (Vilber, Collegien, France). Maximal fluorescence intensity was calculated with ImageJ software after determination of the region of interest. The results are presented as tumor-to-background ration (TBR) where the background was set as the bowel wall proximal to the rectal tumor. Where indicated, TBR was normalized to TBR obtained with free ICG, designated as nTBR.

### Fluorescence microscopy

Fluorescence microscopy analysis was performed on snap-frozen rectal tissues. The tumors were cryo-sectioned at a 10 mm thickness. Frozen sections were fixed at −20 °C acetone, for 7 min, and washed three times for 5 min in PBS. Subsequently, the sections were blocked with 1% BSA for 60 min at RT in a humidified atmosphere. The sections were then incubated overnight at 4 °C with primary anti-CD31-PE antibody (AB-2534231, Ebiosciense, 1:500) and then washed as described above. Mounting medium containing DAPI was then used to mount sections onto coverslips. All images were obtained using a Leica SP8 confocal microscope (Leica Microsystems, Wetzlar, Germany). The H&E sections were analyzed and photographed in an Olympus Ch30 microscope (Tokyo, Japan).

### Data analysis

Data are presented as means ± standard errors (SE). Inter-group differences were calculated using one-way ANOVA with Tukey’s HSD as a post-hoc test. p < 0.05 was considered significant (SPSS for Windows, v25, Munich, Germany).

## Supplementary information

Supplementary information
