# Supplementary material for: Size and lipid modification determine liposomal Indocyanine green performance for tumor imaging in a model of rectal cancer
Source: Sci Rep. 2019 Jun 12;9:8566. doi: 10.1038/s41598-019-45038-w (PMC6561977; doi:10.1038/s41598-019-45038-w)
Supplement: Supplementary file 1 — Supplementary information [file 41598_2019_45038_MOESM1_ESM.docx]

Size and lipid modification determine liposomal Indocyanine green performance for tumor imaging in a model of rectal cancer

Shoshi Bar-David^1^, Liraz Larush^2^ , Noam Goder^1^, Asaf Aizic^3^, Ehud Zigmond^4^, Chen Varol^4,5^, Joseph Klausner^1^, Shlomo Magdassi^2^, and Eran Nizri^1*^

Supplementary Figure S1.


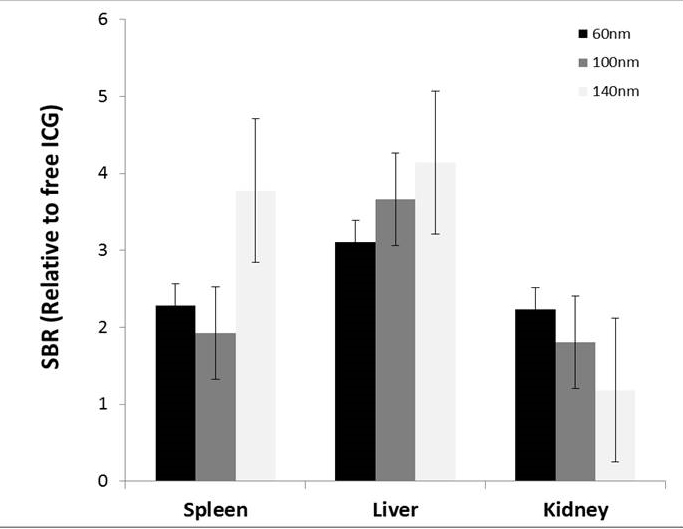


Supp. Fig. S1. Signal intensity of spleen, liver and kidney with various-sized liposomes. Organs were resected and placed in 24-well plates for fluorescence measurement. The results are presented as signal-to-background ratio (SBR), where the background is defined as the same organ of mice injected with free ICG. All measurements were made 12 hours after injection, at the same time point where the tumor signal was measured. The one-way ANOVA for comparison between different sizes showed that the SBR was comparable for each organ (p=0.43, 0.82, 0.76 for spleen, liver and kidney respectively). The results are the summary of two experiments, n=4 for each size.

Supplementary Table S1:

| Total ICG preparation for each experiment (according to 8mg/kg ICG, each mice=0.025 kg): | | | | | | | | |  | |  | |  | |  | |  | |
| --- | --- | --- | --- | --- | --- | --- | --- | --- | --- | --- | --- | --- | --- | --- | --- | --- | --- | --- |
| 0.2mg ICG/mice*11=2.2 mg/experiment=0.0022 gr/experiment | | | | | | | | | | | | |  | |  | |  | |
| Calculation of required dose for each preparation: | | | | | | | | | | |  | |  | |  | |  | |
| **Peg-Lip-ICG** | | | | |  | |  | |  | |  | |  | |  | |  | |
| Total dispersion content | | | | |  | | Solid content | | | |  | |  | |  | |  | |
| Sucrose (gr) | | | 1.74 | |  | | Sucrose (gr) | | 1.74 | | | |  | |  | |  | |
| PEG (gr) | | | 0.9355 | |  | | PEG (gr) | | 0.9355 | |  | |  | |  | |  | |
| Buffer (gr) | | | 16.0351 | |  | | Sucrose (gr) | | 6.99 | | | |  | |  | |  | |
| Sucrose (gr) | | | 6.99 | |  | | Lecithin (gr) | | 3.742 | |  | |  | |  | |  | |
| Lecithin (gr) | | | 3.742 | |  | | Total: | | 13.4075 | |  | |  | |  | |  | |
| Buffer (gr) | | | 64.43 | |  | |  | |  | |  | |  | |  | |  | |
| Total: | | | 93.8726 | |  | |  | |  | |  | |  | |  | |  | |
|  | | |  | |  | |  | |  | |  | |  | |  | |  | |
| Solid content for 70 gr of dispersion taken for the experiment (as described in the Methods section) | | | | |  | |  | |  | |  | |  | |  | |  | |
| 70/93.8276= | | | 0.7456915 | |  | |  | |  | |  | |  | |  | |  | |
| 0.74569*13.4075= | | | | | 9.997859 | |  | |  | |  | |  | |  | |  | |
|  | |  | | |  | |  | |  | |  | |  | |  | |  | |
| After addition of 14gr ICG solution (0.0672gr of ICG): | | | | | | | | | | |  | |  | |  | |  | |
| 9.997859+0.0672 | | | | | 10.065 | | Total solid content | |  | |  | |  | |  | |  | |
|  | |  | | |  | |  | |  | |  | |  | |  | |  | |
| ICG content in each solid gram: | | | | | | |  | |  | |  | |  | |  | |  | |
| 0.0672/10.065= | | | | | 0.00667 | |  | |  | |  | |  | |  | |  | |
| Powder needed for each experiment (0.0022 gr ICG): | | | | | | | | | | |  | |  | |  | |  | |
| 0.0022/0.0067= | | | | | 0.330gr | |  | |  | |  | |  | |  | |  | |
| 330mg of PEG-Lip-ICG powder were dissolved in 2.2 ml buffer and each animal injected with 0.2ml | | | | | | | | | | | | | | | | | | |
|  | |  | |  | |  | |  | |  | |  | |  | |  | |  |
|  | |  | |  | |  | |  | |  | |  | |  | |  | |  |
| **Lip-ICG** | |  | |  | |  | |  | |  | |  | |  | |  | |  |
| Total dispersion content: | | | | | | Solid content: | | | |  | |  | |  | |  | |  |
| Sucrose (gr) | | 18.6 | | | | Sucrose (gr) | | 18.6 | |  | |  | |  | |  | |  |
| Lecithin (gr) | | 10 | |  | | Lecithin (gr) | | 10 | |  | |  | |  | |  | |  |
| Buffer (gr) | | 171.4 | |  | | Total: | | 28.6 | |  | |  | |  | |  | |  |
| Total: | | 200 | |  | |  | |  | |  | |  | |  | |  | |  |
| Solid content for 190 gr of the dispersion used (See methods section): | | | | | | | |  | |  | |  | |  | |  | |  |
| 190/200= | 0.95 | | |  | |  | |  | |  | |  | |  | |  | |  |
| 0.95*28.6= | 27.17 | | |  | |  | |  | |  | |  | |  | |  | |  |
| ICG content: | | | |  | |  | |  | |  | |  | |  | |  | |  |
| 480mg ICG/100 gr | 182.496 for 38.02gr | | |  | |  | |  | |  | |  | |  | |  | |  |
| Solid content after ICG addition: | | | | | |  | |  | |  | |  | |  | |  | |  |
| 27.17+0.182496= | | | | 27.352 | |  | |  | |  | |  | |  | |  | |  |
| ICG content in each solid gram: | | | | | |  | |  | |  | |  | |  | |  | |  |
| 0.182/27.352= | | | | 0.006654 | |  | |  | |  | |  | |  | |  | |  |
| Powder needed for each experiment (0.0022 gr ICG): | | | | | | | | | |  | |  | |  | |  | |  |
| 0.0022/0.006654= | | | | 0.330gr | |  | |  | |  | |  | |  | |  | |  |
|  |  | | |  | |  | |  | |  | |  | |  | |  | |  |
| 330mg of Lip-ICG powder were dissolved in 2.2 ml buffer and each animal injected with 0.2ml | | | | | | | | | | | | | | | | | |  |
